# Deployment and testing of an automated medical equipment service communication and documentation system at a rural hospital in Kenya

**DOI:** 10.1093/inthealth/ihaa103

**Published:** 2021-03-05

**Authors:** Rayonna M Gordon, Santiago J Saldana, Philip J Brown, F Scott Gayzik, Evelyn Y Anthony

**Affiliations:** Center for Injury Biomechanics, Wake Forest University, Winston Salem, NC 27101, USA; School of Medicine, Wake Forest University, Winston Salem, NC 27101, USA; Public Health Sciences, Wake Forest University, Winston Salem, NC 27101, USA; Center for Injury Biomechanics, Wake Forest University, Winston Salem, NC 27101, USA; School of Medicine, Wake Forest University, Winston Salem, NC 27101, USA; Center for Injury Biomechanics, Wake Forest University, Winston Salem, NC 27101, USA; School of Medicine, Wake Forest University, Winston Salem, NC 27101, USA; School of Medicine, Wake Forest University, Winston Salem, NC 27101, USA

**Keywords:** documentation, equipment failure, maintenance, point-of-care systems

## Abstract

**Background:**

The Medical Equipment Network Documentation System (MENDS) provides a simple communication network for equipment servicing from failure to restoration. It is a text messaging-based platform, designed to use existing technologies in place in low- and middle-income settings. The system gathers and relays information about equipment service requests and reports and automatically saves them to an online database.

**Methods:**

MENDS was deployed at a high volume, rural, charity medical facility in Kijabe, Kenya for a 3-mo pilot test.

**Results:**

The results show MENDS more than tripled documentation and enhanced ease and speed of communication.

**Conclusions:**

Comprehensive data provided by MENDS created more accurate measures of equipment performance, which can be used to decrease the time that equipment is out of service and improve the efficiency of repairs, equipment quality and procurement.

## Introduction

Medical facilities in low- and middle-income countries (LMICs) experience difficulties with their medical equipment due to resource hindrances including a shortage of trained technical staff to properly operate and maintain the equipment, as well as unavailability of reliable and continuous power and water sources.^1^ These barriers contribute to equipment failures and impede proper repair, often leaving the equipment to lie unused for long periods of time, disrupting medical professionals’ ability to care for their patients and draining the facility's finances.

This operating environment was studied during an intensive investigation at AIC Kijabe Hospital (KH) in Kijabe, Kenya in December 2018. KH is a 363-bed non-profit mission medical facility in a rural part of southwestern Kenya. It has numerous inpatient and outpatient (OP) services, a highly specialized surgical department and several satellite clinics.^2^ KH is also a referral and teaching hospital providing training for medical students from several countries.^2^ Since KH is a charity facility, the costs for its operation and services are subsidized by affiliate churches requiring a small copay from patients.^3,4^ There are over 900 staff at the KH Kijabe location made up of Kenyan natives and missionaries from around the world. This campus has a number of departments including Emergency, Ear, Nose and Throat, Pediatrics, Intensive Care Unit (ICU), OP and Palliative Care.^5^ Equipment from these departments vary from autoclaves, incubators and ventilators to oxygen tanks, patient monitors and patient beds, with varying brands and models like General Electric and Siemens as well as local Kenyan brands such as Meximed. KH sees over 180 000 patients per year just in OP services and many of the departments are open 7 d per week.^6^

The challenges around documentation of equipment services include effective communication, time management and accountability. This was observed by a member of the study team (RMG) at KH in preparation for and execution of this study. The current organization and communication structures for equipment maintenance for many medical facilities in Kenya, including KH, are either manual or a computer program. In the manual method, equipment information and services are communicated verbally and handwritten, which can be inconsistent and leave room for error. The computer program method requires information to be logged into an online portal, which is time-consuming and not easily accessible. An effective system would need to be quick, consistent and easily accessible while including key factors such as maintenance records, well-structured and centrally located documentation, as well as regular reports on equipment status regarding finances and staff performance.^7^

This study focused on the deployment and onsite trial testing of an established automated communication network called the Medical Equipment Network Documentation System (MENDS) for equipment servicing from failure to restoration in a LMIC. MENDS’ details and operation were formulated by extensive input from the KH Biomedical Engineering Department (Biomed). This study addressed the supervisory and system capacity of medical facilities in the chain of systemic capacity. Supervisory and system capacity pertain to the ability to report and monitor, as well as the effective and timely flow of information.^8^ This study was a pragmatic deployment establishing a basis for MENDS and its usefulness. The goal was to evaluate the impact of MENDS at KH, to obtain a more comprehensive and organized record of equipment services and to improve communication and responsiveness, e.g. the speed at which the issue was addressed.

## Materials and Methods

### Current manual method

Currently KH communicates requests for equipment service by telephone call. When equipment malfunctions, users call engineers on their personal mobile phones or the Biomed landline. The user explains the issue to the engineer who then goes to the department to tend to the request. Once the engineer completes the work, they manually record the details of the request and the service into a notebook. Those details are eventually transferred to a Microsoft Excel (Microsoft Corp., Redmond, WA, USA) spreadsheet to create a master log. The data logged in the Excel spreadsheet were analyzed and evaluated against the MENDS data from the pilot test and the findings are revisited in the Results and Discussion sections of this study. The analysis includes evaluating user and engineer input and comparing the number of documented equipment service entries.

### MENDS system design

MENDS was developed to capture and document equipment service requests and repairs. To ensure compatibility with the developing infrastructure, MENDS utilizes text messaging to communicate information rather than a mobile application. Only about 26% of the Kenyan population use the internet; however, 97% have mobile phones, making them the ideal platform.^9^ MENDS has three components: the PHP (Zend Technologies, Cupertino, CA, USA), MySQL (Oracle Corporation, Austin, TX, USA), jsGrid/JavaScript (Oracle Corporation, Austin, TX, USA) code written to run MENDS automation, the short message service application programming interface (SMS API) to allow for the use of text messaging as the form of communication and a developer cloud to hold all the information captured.

MENDS was adapted to function in Kenya using Africa's Talking Ltd (Lavington Nairobi, Kenya) for the SMS API to ensure quick, local communication. A dedicated shortcode was purchased at 11 600 Kenyan Shilling (KSH) with a 17 000 KSH per month maintenance fee (US${\$}$108.95 and US${\$}$159.67, respectively). The cost for MENDS to send messages to users was 0.70 KSH per outgoing message (US${\$}$0.0066). Purchasing the dedicated shortcode gave MENDS two-way communication capabilities and avoided users needing to pay text messaging costs while communicating with MENDS. This cost avoidance was especially important as users would primarily be using their own personal mobile devices. The information MENDS collected was backed up to an online cloud server using Digital Ocean (New York, NY, USA) routed through Bangalore, India. This created an ongoing, continuously updated database. This database can be accessed by logging into a password-protected website to provide a layer of security. Security needs were determined as minimal by the team and KH administration because the data would not contain any sensitive hospital or patient information.

**Figure 1.**
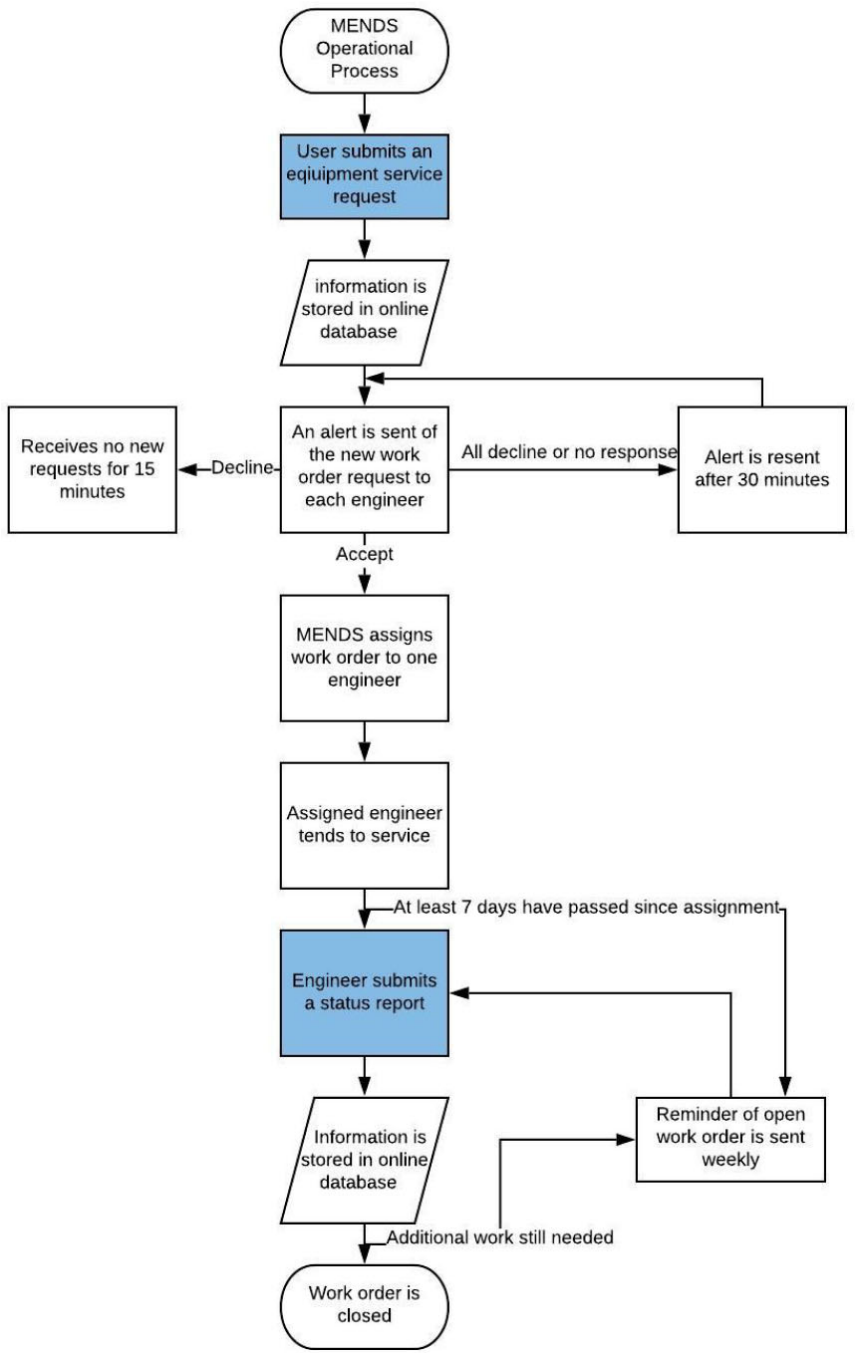
Flowchart of Medical Equipment Network Documentation System (MENDS) operation and information.

MENDS’ operation is briefly described in its three functions: requesting, assigning and reporting. The flow of the process is seen in Figure 1. When equipment malfunctions, equipment users submit a request for a service via text message to MENDS, including the equipment type, department location, specific equipment issue, urgency and user's name. MENDS sends a response to the user, restating the submission details for user verification. Once verified, the information of the request is saved to the online database. MENDS then sends a text alert to each engineer to inform them of the details of the request and to ask them to either accept or decline the work order. The engineers have 5 min following the alert to respond. After 5 min, MENDS assigns the work order to one of the accepting engineers. Assignment selection is based on the availability of engineers and the number of outstanding work orders to ensure work is distributed equitably. The assigned engineer is responsible for managing the service and completing a status report.

**Figure 2.**
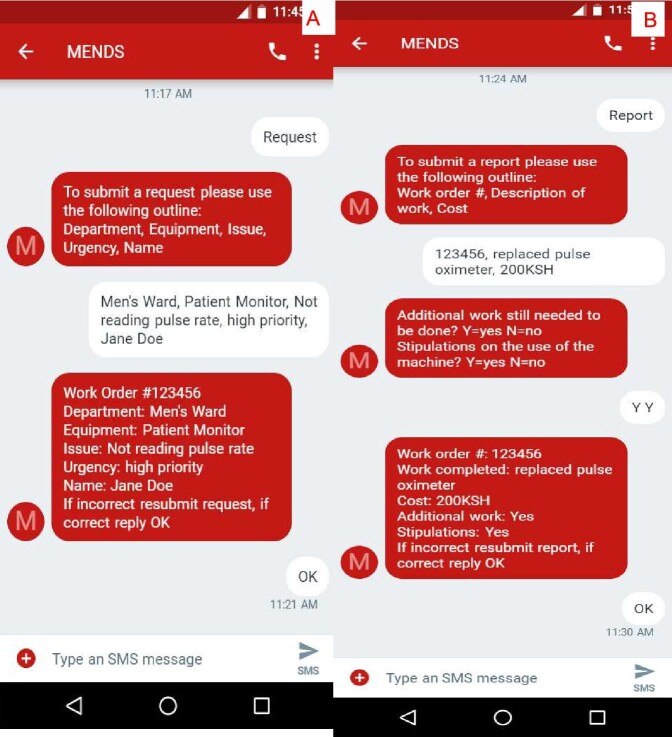
Text message conversations with MENDS. (A) user requesting service and (B) engineer reporting service.

Engineers are responsible for contacting MENDS when work is complete via text message, detailing the work order number, a description of the work completed, the cost associated with the service, any need for additional work on the equipment and stipulations regarding the use of the equipment. MENDS sends a response to the engineer restating the submission details for verification. Once verified, the report information is saved in the online database and the work order is closed. Weekly reminders are sent to each engineer with the number of the work orders that are open. An example of a text message conversation is provided in Figure 2. This design was tested in Winston Salem, North Carolina at Wake Forest Baptist Medical Center to ensure the system worked as intended and to optimize its functionality.

### Pilot testing method

#### Deployment strategy

Testing of MENDS was performed by deployment and pilot usage at KH. Participants in the study included the five engineers responsible for the maintenance of all the medical equipment at the facility and the 24 staff across each participating department responsible for reporting equipment service needs. The users, the staff responsible for reporting, varied in quantity and title for each department but included the department supervisors. Prior to receiving instructions on using MENDS, each participant was asked to complete a survey to obtain their opinion on the current manual equipment maintenance documentation method.

Next, users in each participating department received, in person, instructions on how to use MENDS and how to locate further support resources if necessary. The engineers received more in-depth training on operating MENDS both as a user and as an engineer. Following the survey and presentation, each user and engineer was instructed to test MENDS on their own personal devices. They were guided through the process and all questions were answered to ensure successful repetition throughout the testing period. The instructional period lasted 3 d, with visits to each department. Once a department received their training, they were instructed to begin using MENDS immediately, so after 3 d all the participating departments were using MENDS. Five additional days were allocated for troubleshooting.

The pilot test ran for 3 mo from February to April 2020 and included the following departments: OP, Casualty (emergency), Pediatrics, Dental, Theatre (operating rooms), ICU, satellite clinics, inpatient and maternity wards and laboratories. Afterwards, users and engineers were once again surveyed about their experiences with MENDS.

#### Data gathering

Data were gathered using two sources, participant surveys and equipment service information from the online database. All users and engineers were given a paper survey at the beginning of their training session. They were given time to complete the survey before instruction began. The surveys asked them to specify the method of communicating and documenting equipment services, how often they made service requests and reports, as well as the benefits and issues they experienced with the method. The surveys also asked for a rating from 1 (strongly disagree) to 5 (strongly agree) on several aspects of the method including satisfaction, efficiency, ease, comfort and effectiveness. All 29 surveys were collected upon completion. Following the 3-mo testing period, the same survey was given to all users and engineers via email or text message in the form of a Google Form. Participants were given 2 wk to complete and return the survey. Surveys were received from seven users and four engineers. The distribution of the surveys following the test was changed from paper to a digital format due to the inability to travel back to KH because of the travel restrictions caused by the COVID-19 pandemic.

MENDS automatically saved all information onto an online database. This information included department, equipment type, a description of the issue the equipment experienced, the urgency of the issue, time and date the request was submitted, a description of the service performed on the equipment, cost of the services, as well as the time and date the report was submitted. Following the testing period, the information from the database was copied and placed into the statistical analysis engines Excel and MatLab. The same information, excluding urgency, was retrieved in the raw data from the Excel master log collected using the manual method from February to April in 2018 and 2019. This information was captured by KH Biomed and sent directly to the team for this study.

#### Evaluation method

Survey data were evaluated by comparing the responses of the pretest survey, input via the current manual method, with those of the post-test survey, which were input via MENDS. Since the pre- and post-test survey questions were the same, a direct comparison was able to be made aligning overall and individual responses. Only surveys from participants who completed both the pre- and post-test survey were included in the evaluation. All survey information was used to determine the effect on communication, time management and responsiveness of parties involved in the equipment maintenance process. Equipment service data were evaluated by comparing the quantity of services documented by the manual method and MENDS to quantify the organization. Data were also analyzed by the performance measurements that were able to be made to evaluate the quality of the equipment and services and to quantify comprehensiveness and accountability. These measurements were made by taking the raw data from the MENDS database and analyzing their components across departments and time.

## Results

MENDS was used for a total of 3 mo by 10 departments at KH as well as Biomed. Surveys were given only to engineers and to users whose duties included requesting equipment service needs. The participants, from which both pre- and post-test surveys were received, aged from 23 to 52 y and consisted of nine males and two females. Their job titles ranged from Department Manager to Assistant Nurse to Technician.

### Equipment maintenance results

**Figure 3.**
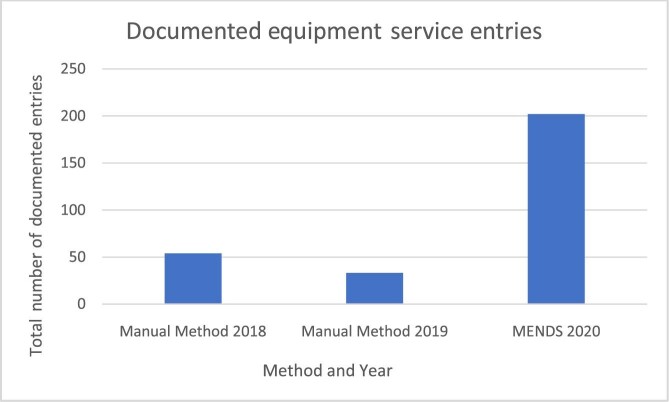
Total number of documented equipment service entries using the manual method in 2018 and 2019 vs MENDS in 2020 across the 3-mo period.

To quantify organization of the maintenance systems, the number of documented equipment services was measured to determine how much was being captured with each method. This measurement was the only available accurate comparison between the manual method data and MENDS data. Figure 3 displays the total number of equipment service entries documented, including completed and open work orders, for the manual method and MENDS. Documentation is from February to April for the manual method in 2018 and 2019 and for MENDS in 2020. MENDS chronicles more than three times as many entries than the manual method.

### Equipment performance results

**Figure 4.**
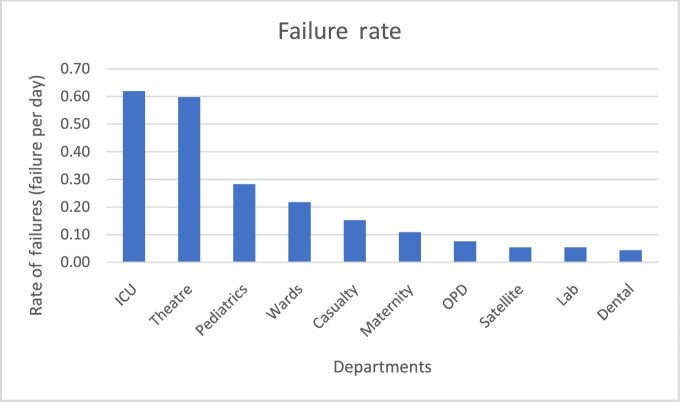
The equipment failure rate in each department using MENDS within the 3-mo testing period.

To quantify the effect on comprehensiveness and accountability, performance metrics were calculated from data collected using MENDS to determine if useful information was being captured. All measurements include completed and open work orders. Figure 4 shows the failure rate of equipment per department while using MENDS. Failure rate is the probability per time that the department experiences equipment failure throughout the testing period. The equation for failure rate is:

$$\begin{eqnarray*}
{\rm Failure \ Rate} = {Number\,of\,failures/ Total\, test\,\, period \, time\,\, \left( {3 \ {\rm mo}} \right)}
\end{eqnarray*}$$

The highest failure rate was in the ICU and Theatre departments, showing over 0.6 failures per day.

**Figure 5.**
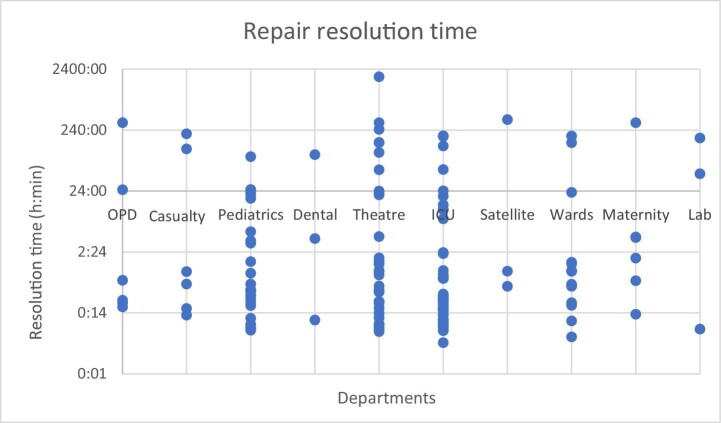
Resolution time of services (time from submission of request to the submission of its corresponding report) in each department when using MENDS within the 3-mo testing period.

Figure 5 shows the resolution time of each service in each department over the testing period. Time is specified in hours and minutes on a logarithmic scale; e.g. 0:01 = 1 min; 24:00 = 24 h; 2400:00 = 2400 h = 100 d. Resolution time is the time from the submission of a service request to the submission of its corresponding report. There was a wide variety of resolution times in each department with the shortest being <14 min and the longest being >10 d. The results of a Kruskal-Wallis H test showed a p-value of 0.4603, demonstrating that there was no significant difference in resolution time between departments.

### Survey results

Survey results were used to evaluate the effect on communication, time management and responsiveness. Eight of 11 surveyed participants specified either a neutral response or disagreement with overall satisfaction with the manual method. Ten of 11 surveyed participants whose original satisfaction response regarding the manual method was ‘disagree’, ‘neutral’ or ‘agree’, communicated greater satisfaction with MENDS by strongly agreeing with ‘overall satisfaction’.

Table 1 displays average ratings, range of the ratings and % increase from the manual method to MENDS for the remainder of the statements in the pre- and post-test surveys from the users. The rating scale goes from a minimum of 1 (strongly disagree) to a maximum of 5 (strongly agree). Ratings were 4.9 or higher when pertaining to statements about MENDS, while statements pertaining to the manual method received a highest rating of 3.9. Statements relating to communication—the ability to effectively request a service, system efficiency and learning how to use the system—showed at least a 37% increase in rating from the manual method to MENDS, with all survey participants giving a rating of 5.0 for MENDS. Statements relating to time management—ease of use and the ability to quickly request—showed at least a 31% increase in rating, with one survey participant giving a rating of <5.0 for MENDS. Comfortability had the greatest % increase in rating at 108%.

**Table 1.** Average ratings, range of ratings and % increase for users’ pre- and post-test surveys regarding the manual method and the Medical Equipment Network Documentation System (MENDS)

	Average rating (out of 5)		Range		
User	Manual method	MENDS	Manual method	MENDS	Increase (%)
Overall, I am satisfied with the system	3.1	5.0	2	0	73
The system is easy to use	3.9	5.0	1	0	31
I am able to quickly request equipment service	3.6	4.9	2	1	45
I am able to effectively submit a request for equipment service	3.1	5.0	2	0	79
It was easy to learn how to use the system	3.7	5.0	1	0	37
The system is efficient	3.3	5.0	3	0	69
I feel comfortable using the system	3.0	5.0	3	0	108

Table 2 displays average ratings, range of the ratings and % increase from the manual method to MENDS for the remainder of the statements in the pre- and post-test surveys from the engineers. Ratings were 4.5 or higher when pertaining to statements about MENDS, while statements pertaining to the manual method peaked at 3.5. Statements relating to communication—the ability to effectively report a service, system efficiency and learning how to use the system—showed at least a 54% increase in rating from the manual method to MENDS, with a 50% decrease in variability in responses. Statements pertaining to time management—ease of use and receiving requests, quickness of assigning requests and ability to quickly report—showed at least a 46% increase in rating, with one survey participant giving a rating of <5.0 for MENDS.

**Table 2.** Average ratings, range of ratings and % increase for engineers’ pre- and post-test surveys regarding the manual method and the Medical Equipment Network Documentation System (MENDS)

	Average rating (out of 5)		Range		
Engineer	Manual method	MENDS	Manual method	MENDS	Increase (%)
Overall, I am satisfied with the system	2.8	4.8	1	1	79
The system is easy to use	3.0	5.0	2	0	77
I am able to easily receive new equipment service requests	3.0	5.0	2	0	88
New service requests are assigned quickly and fairly	3.0	4.8	2	1	71
I am able to quickly report equipment service	3.5	5.0	1	0	46
I am able to effectively submit a report for equipment service	2.8	5.0	1	0	88
It was easy to learn how to use the system	3.3	4.5	2	1	54
The system is efficient	2.8	4.8	2	1	90
I feel comfortable using the system	3.0	4.8	2	1	69

Table 3 lists the overall themes of the advantages and disadvantages for each system from the free text comments conveyed by participants in the surveys. Many of the disadvantages with the manual method, such as responsiveness, recordkeeping, ownership and efficiency of requesting and reporting, were advantages for MENDS.

**Table 3.** Comments taken from users’ and engineers’ pre- and post-test surveys regarding the manual method and the Medical Equipment Network Documentation System (MENDS)

Manual method	MENDS
Advantages	
Direct contact with engineers	Easy to contact engineers and quick response Quick, simple and easy request and report submission Comprehensive records Efficient ownership
Disadvantages	
Difficulty contacting engineer and delay in response Lack of records Poor ownership and accountability Inefficient request and report submission	Difficulty to quickly learn how to use Incorrectly used

It should be noted that 18 out of 27 people trained to use MENDS completed at least one request or report, yielding a 66% adoption rate. More than 50 people in the participating departments who did not receive official formal training were able to learn and use MENDS over the course of the 3-mo testing period.

## Discussion

This study describes the deployment and 3-mo testing of an automated equipment maintenance system, MENDS, at KH in Kenya. Within 3 d, MENDS was deployed at KH, and over the course of the 3 mo, it provided improved communication between equipment users and engineers as well as increased accountability of engineers; it more than tripled the documentation of services and increased the comprehensiveness of documented information.

The current manual equipment maintenance method is varied and unpredictable; consequently, it is necessary to rely heavily on qualitative data from participant surveys to evaluate its effect upon communication. The records from using the manual method were deemed unreliable for several reasons. A significant increase of equipment service entries was documented when using MENDS vs the manual method. This finding suggests that the manual method under-recorded equipment services. Under the manual method, there was no documentation for open work orders, meaning that all documentation was carried out on completed work orders only. The timestamps documented on each entry using the manual method may be incorrect because they were made manually; information about the request and the completion report were documented simultaneously when the work was completed; several entries showed that the completion report was dated earlier than the request (in those cases it was assumed that the times were transposed); and for several entries the request and completion report time/date were recorded as being the same.

It is important to note that participant input is crucial to determining the success of MENDS. The input and opinion of the users and the engineers regarding the benefit and ease of using a new system testifies to their readiness to adopt it. Without their participation and endorsement, any new method will be useless, whatever its strengths.

### Equipment maintenance results

#### MENDS response to organization

As captured in Figure 3, MENDS resulted in a substantial increase in the number of equipment services documented. This confirms successful communication, service completion and documentation using MENDS. The ability of MENDS to collect a larger number of documents proves that it was successful in streamlining the flow of information and organizing the maintenance process. It was able to better reflect the volume of services being carried out at KH.

### Equipment performance results

#### MENDS response to comprehensiveness and accountability

MENDS gives a more accurate depiction of the performance and maintenance of equipment in each department at KH. Figures 4 and 5 reveal the unreliable nature of the equipment. In Figure 5, common issues were identified among services with the longest and shortest resolution times. Longer resolution times often stemmed from a need to obtain the equipment manual or to travel to obtain spare parts. These processes are known to cause significant delays. The shorter resolution times occurred when equipment was not broken but simply needed a service, such as refilling oxygen tanks or repositioning equipment. Both of these services can be performed quickly and onsite.

Overall, the data received from MENDS more accurately align with the testimonies and observations made at KH and with published research regarding the condition of medical equipment in LMICs. This additional information obtained from MENDS provides comprehensive quantitative evidence regarding items of equipment, thus enabling tracking to pinpoint areas for improvement while also establishing accountability.

### Survey results

#### MENDS response to communication, time management and responsiveness

The survey results in Tables 1 and 2 suggest there is a gap in effective communication when using the manual method. The increased ratings with MENDS indicate that it is able to bridge that gap. The survey results confirm the ability of MENDS to make the communication process for equipment service requesting, assigning and reporting quick, efficient and effective. Comments in Table 3 further support MENDS as a viable tool for communication and documentation. MENDS improved responsiveness and ownership among the engineers while being simple, easy and time-saving. More than 50 personnel within the departments who did not receive formal training were able to use MENDS easily and correctly, demonstrating that MENDS is user-friendly.

### Limitations

This study has several limitations in addition to the variable data obtained from manually created records. First, a few participants incorrectly texted when sending information to MENDS. Second, it was impossible to track individual equipment items because there was no existing labeling system. For example, KH has multiple ventilators but they could not be individually tracked. Third, the dedicated shortcode cost is per mobile network, so we only paid for connectivity with Safaricom PLC (Nairobi, Kenya) as most personnel at KH were service users with Safaricom. Therefore, only users with Safaricom mobile plans were able to communicate with MENDS. Also, the cost of the pilot test was covered by the research team, necessitating a 3-mo window to gather data. This short study gave us the ability to collect pilot data on MENDS as it is a novel concept; however, a longer data collection period is necessary to determine sustainability. Finally, the 2019 COVID-19 pandemic began in Africa in the middle of this study. KH had to adapt its services to the infectious diseases environment, which altered its use of equipment and its servicing requirements. Moreover, travel to Kijabe to complete the pilot test in person was rescinded.

### Conclusions

Data collected from the 3-mo pilot test, pre- and post-test surveys, additional written documentation from participants and first-hand observations all indicate that MENDS successfully met the study goal. MENDS provided a user-friendly, adaptable platform that improved communication by increasing ease and speed, as well as increased quantity and quality of documentation. The consistent and accurate data allowed for supplementary measurements to analyze equipment performance. Although the manual method was operated at little or no cost for the facility and MENDS operates at about 234 000 KSH per year (US$2200), the ability of MENDS to capture a higher quantity and quality of data results in greater long-term savings. These benefits include the ability to monitor equipment and predict when it will need replacing, increased efficiency of repairs for engineers and decreased resolution time, returning equipment to service and increasing patient care and throughput. MENDS provides the evidence necessary to predict when current devices are end-of-life so that the facility can plan for future purchases or readily inform potential donors about equipment types that are required.

We plan to continue testing MENDS to collect additional data to evaluate trends, sustainability and participant retention. Although this study only entailed using MENDS for 3 mo, all of the participants requested its continuation and are currently using MENDS. Further improvements to MENDS include minimizing the cost of its operation by sharing the dedicated shortcode among multiple facilities. Other improvements consist of incorporating a preventative maintenance schedule and creating an equipment inventory database, with tracking of individual pieces of equipment, especially the more expensive items. The end result is a system that will lower overall equipment costs and allow items to be repaired more quickly and efficiently, thereby enabling important medical facilities to meet the healthcare needs of their local populations.

## Data Availability

The authors confirm that the data supporting the findings of this study are available within the article. Raw data are not publicly available due to healthcare security and participant confidentiality purposes. Additional data analysis is available upon request with permission from KH personnel.
